# Improvement of Left Ventricular Function under Cardiac Resynchronization Therapy Goes along with a Reduced Incidence of Ventricular Arrhythmia

**DOI:** 10.1371/journal.pone.0048926

**Published:** 2012-11-12

**Authors:** Christian Eickholt, Marcus Siekiera, Kiriakos Kirmanoglou, Astrid Rodenbeck, Nicole Heussen, Patrick Schauerte, Artur Lichtenberg, Jan Balzer, Tienush Rassaf, Stefan Perings, Malte Kelm, Dong-In Shin, Christian Meyer

**Affiliations:** 1 Heinrich-Heine-University Duesseldorf, Medical Faculty, Department of Cardiology, Pulmology and Angiology, Dusseldorf, Germany; 2 Division of Cardiology, Pulmology and Angiology, University Hospital RWTH-Aachen, Aachen, Germany; 3 Department of Medical Statistics, University Hospital RWTH-Aachen, Aachen, Germany; 4 Heinrich-Heine-University Duesseldorf, Department of Cardiovascular Surgery, Dusseldorf, Germany; Tokai University, Japan

## Abstract

**Objectives:**

The beneficial effects of cardiac resynchronization therapy (CRT) are thought to result from favorable left ventricular (LV) reverse remodeling, however CRT is only successful in about 70% of patients. Whether response to CRT is associated with a decrease in ventricular arrhythmias (VA) is still discussed controversially. Therefore, we investigated the incidence of VA in CRT responders in comparison with non-responders.

**Methods:**

In this nonrandomized, two-center, observational study patients with moderate-to-severe heart failure, LV ejection fraction (LVEF) ≤35%, and QRS duration >120 ms undergoing CRT were included. After 6 months patients were classified as CRT responders or non-responders. Incidence of VA was compared between both groups by Kaplan-Meier analysis and Cox regression analysis. ROC analysis was performed to determine the aptitude of LVEF cut-off values to predict VA.

**Results:**

In total 126 consecutive patients (64±11years; 67%male) were included, 74 were classified as responders and 52 as non-responders. While the mean LVEF at baseline was comparable in both groups (25±7% vs. 24±8%; P = 0.4583) only the responder group showed an improvement of LVEF (36±6% vs. 24±7; p<0.0001) under CRT. In total in 56 patients VA were observed during a mean follow-up of 28±14 months, with CRT responders experiencing fewer VA than non-responders (35% vs. 58%, p<0.0061). Secondary preventive CRT implantation was associated with a higher likelihood of VA. As determined by ROC analysis an increase of LVEF by >7% was found to be a predictor of a significantly lower incidence of VA (AUC = 0.606).

**Conclusions:**

Improvement of left ventricular function under cardiac resynchronization therapy goes along with a reduced incidence of ventricular arrhythmia.

## Introduction

Cardiac resynchronization therapy (CRT) has become an integral component of systolic heart failure (HF) therapy. Up to date there is a bulk of evidence for the beneficial effect of CRT in patients with moderate-to-severe clinical impairment, which includes improved quality of life, fewer hospitalizations and decreased mortality [1]. The antiarrhythmic effects of CRT are well known [2]–[4]. However there are also reports of proarrhythmic effects [5]–[7]. Intriguingly a meta-analysis of large clinical trials, involving patients being implanted with CRT-devices with defibrillator backup (CRT-D), could not show a reduction in device interventions due to ventricular arrhythmias (VA) [8]. Also despite careful patient selection and elaborate efforts in post-implantation management CRT is only successful in about 70% of patients [9]. In patients responding to CRT left-ventricular reverse remodeling leads to a reduction of myocardial stretch and favorable neurohumoral changes [10], [11]. Furthermore it can invoke profound changes on the (sub)cellular level [12], [13]. Those effects might translate into a stabilizing effect on cardiac electrophysiology [14], [15]. Besides reduction in left-ventricular end-systolic volume (LVESV), as the established marker for mechanical remodeling, improved left ventricular systolic function (LVEF) could be a functional and easily obtainable alternative, which might be less afflicted by inter- and intra-observer variability [16]–[19]. Importantly, previous studies demonstrated that among CRT responders there is a decrease in VA [20]. However, whether an improvement of LVEF among CRT patients goes along with a decreased incidence of VA has not been prospectively evaluated.

To address the impact of an improved LV function on the occurrence of ventricular arrhythmias we analyzed the incidence of VA in CRT responders in comparison with non-responders.

## Methods

### 1. Patients

126 consecutive heart failure patients who underwent implantation with a combined CRT and cardioverter-defibrillator device (CRT-D) were included. Eligibility for CRT was based on 1) moderate-to-severe HF (NYHA functional class III or IV) despite optimal conventional therapy, 2) LVEF ≤35% as demonstrated by echocardiographic assessment and 3) left bundle branch pattern on the electrocardiogram (ECG) with a QRS duration >120 ms [21], [22].

This study was conducted with approval of the ethics review committees (ERC) appointed by each centre (Heinrich-Heine-University Duesseldorf: ERC of the medical faculty, Building 13.41, Moorenstrasse 5, 40225 Duesseldorf; RWTH Aachen: ERC of the medical faculty, Pauwelstrasse 30, 52074 Aachen). Data collection for scientific purposes was covered under a written consent given by the subjects upon admission to a teaching hospital.

### 2. CRT implantation

CRT systems were implanted as previously described [21], [23], [24]. The LV pacing lead was inserted by a transvenous approach through the coronary sinus into either the lateral or posterolateral cardiac vein whenever possible. All patients who received CRT devices had biventricular stimulation of the heart with right ventricular leads.

### 3. Follow – up

#### 3.1 Device evaluation

All patients were seen for outpatient clinic visits at 4 weeks post CRT implantation and in 3-month intervals after implantation. A physician contacted patients who failed to present to the prescheduled routine visits by phone. All follow-up visits included an interview, physical examination, an ECG, and a device interrogation. From the device printouts incidence and type of arrhythmias were determined by two trained electrophysiologists. Analysis of the printouts was blinded. Shocks or antitachycardic pacing (ATP) were classified as appropriate when they occurred in response to VT or VF and as inappropriate when triggered by sinus or supraventricular tachycardia, T-wave oversensing, or electrode dysfunction [25], [26].

As described before the cycle length of the first ventricular arrhythmia triggering defibrillator (ICD) therapy and the average cut-off rate of the VT detection zone were standardized [27]. VT and VF zones were uniformly programmed with the same cut-offs for primary and secondary prevention patients. A VT-zone with primary application of ATP was programmed for CL from 450 ms to 300 ms, the VF-zone with immediate shock delivery was programmed for CL lower than 300 ms.

#### 3.2 Clinical evaluation

Heart failure symptoms were classified using the New York Heart Association (NYHA) score. Resting 2-dimensional echocardiography was performed at baseline and 6 months follow-up [22]. Biplane LVEF assessment according to Simpson's rule was obtained for each patient in the apical 2- and 4-chamber view [28]. Volumetric parameters were acquired by biplane summation as reliably feasible in the individual [21]. Patients were classified as responders, based on an improvement in NYHA functional class by ≥1 and/or an improvement by ≥10% in LVEF 6 month after device implantation [29], [30].

### 4. Statistical analysis

The primary end point was the occurrence of spontaneous sustained VT or VF leading to appropriate ICD intervention. Continuous data are expressed as mean ± standard deviation (SD). Categorical data are presented by frequencies and percentages. Differences in baseline characteristics and 6-month follow-up were evaluated using unpaired Student t-test and Fisher's exact test as appropriate. Data within patient groups (to compare the effect of CRT) were compared by the use of paired Student t-tests. Survival curves were calculated and graphically presented using the Kaplan-Meier method for censored failure time data. Cox regression analysis was performed to evaluate associations between risk factors at enrollment and appropriate VT/VF therapy. Variable selection process was performed in two steps. Starting with the univariate analysis of the potential association of baseline findings with the occurrence of sustained VT or VF. This step was used as a model building process with factors showing a p-value of less or equal to 0.2 being used in the corresponding multivariate statistical model. The resulting multivariate Cox regression models were stratified by responders, non-responders and ICM or DCM respectively. In this last step, factors were assessed as significant, if the p-value was less than 0.05. ROC analysis was conducted after prior testing by multivariate analysis. All analyses were performed using SAS® statistical software, V9.1.3 (SAS Institute, Cary, NC, USA).

## Results

### 1. Patients

The baseline characteristics of patients are shown in Table 1. There were no differences in clinical baseline characteristics between patients found to be responders or non-responders except for a slightly higher number of patients with secondary preventive implantation in the responder group. The cause of heart failure was ischemic in 65 patients (52%) and non-ischemic in 61 patients (48%).

**Table 1:** Comparison of baseline clinical characteristics between responders and non responders.

	Responders (n = 74)	Non- Responders (n = 52)	p-value
Men, n (%)	49 (66)	36 (69)	0.847
Age, years	64±10	64±12	0.537
Body mass index, kg/m2	28±4	26±4	0.462
QRS duration, ms	160±19	158±24	0.4
NYHA class IV, n (%)	13 (18)	5 (10)	0.209
LVEF, %	25±7	24±8	0.742
ICM, n (%)	34 (46)	31 (60)	0.15
Cardiovascular history, n (%)			
Previous CABG	10 (14)	13 (25)	0.108
Previous PCI	16 (21)	15 (29)	0.404
NSVT	10 (14)	7 (14)	1.0
Aborted SCD	20 (27)	8 (15)	0.134
Rhythm, n (%)			
Sinus rhythm	63 (85)	43 (83)	0.806
Atrial fibrillation	11 (15)	9 (17)	0.806
Comorbidity, n (%)			
Diabetes	30 (41)	14 (27)	0.132
Dyslipoproteinaemia	51 (69)	35 (67)	0.849
Hyperuricaemia	39 (53)	24 (46)	0.587
Hypertension	51 (69)	35 (67)	0.849
Medication, n (%)			
Beta-blockers	67 (91)	48 (92)	1.0
ACE-inhibitors/ARB	73 (99)	49 (94)	0.305
Loop diuretics	62 (84)	48 (92)	0.184
Spironolactone	59 (80)	39 (75)	0.664
Digitalis	40 (54)	28 (54)	1.0
Amiodarone	17 (23)	17 (33)	0.308
Statins	39 (53)	31 (60)	0.471

ICM = ischemic cardiomyopathy; CABG = coronary artery bypass graft; CRT = cardiac resynchronization therapy; ICD = implantable cardioverter-defibrillator; LVEF = left ventricular ejection fraction; NSVT = non-sustained ventricular tachycardia; NYHA = New York Heart Association; PCI = percutaneous coronary intervention; SCD = sudden cardiac. death; All differences between responders and non-responders are statistically not significant.

### 2. CRT response

**Figure 1:**
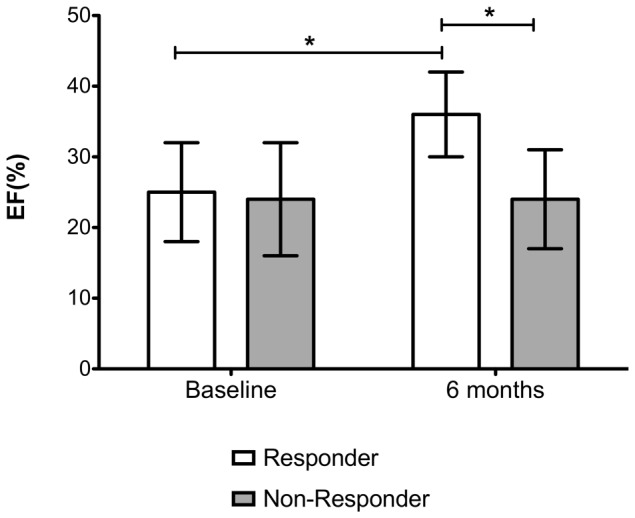
Development of mean left ventricular ejection fraction (EF) in the collectives with and without response to cardiac resynchronization therapy at baseline and at 6 months after device implantation. Error bars indicate standard deviation. While there is no increase in EF in the non-responder group, there is significant improvement in the responder group (* = p<0.0001; see also Table 2).

Mean LVEF increased within 6 month after CRT device implantation from 25±7% to 31±8% (p<0.001), with a reduction in LVESV from 174±73 ml to 138±69 ml (p<0.001). There were 74 (59%) patients, among our collective of 126 individuals, fulfilling the criteria for positive CRT response as established above (Table 1). In the responder group the mean LVEF improved from 25±7% to 36±6% (p<0.001), while in the non-responder group no significant increase occurred (Figure 1). Furthermore, in the responder group a decrease in LVESV from 176±72 ml to 122±68 ml (p<0.001) was noted, while in the non-responder group no significant decrease in LVESV (baseline: 169±73, follow-up: 162±69; p = 0.521) occurred. This also applied for the development of NYHA classes. The baseline NYHA classes were matched between responders and non-responders. At the 6-month interval there was a statistically significant association of CRT-response with development of the NYHA class. In the responder group there was a significant shift of patients from NYHA class IV and III to lower classes (Table 2).

**Table 2:** Comparison of changes in NYHA functional class and left ventricular ejection fraction (LVEF) between responders and non-responders.

		All Patients (n = 126)	Responders (n = 74)	Non-Responders (n = 52)	p value (responders = vs. non-responders)
LVEF					
Baseline		25±7%	25±7%	24±8%	0.458
6 months post CRT		31±8%*	36±6%*	24±7%	<0.001
NYHA class		n (%)	n (%)	n (%)	
Baseline	NYHA I	0 (0)	0 (0)	0 (0)	fisher's exact testp = 0.152
	NYHA II	9 (7)	3 (4)	6 (12)	
	NYHA III	99 (79)	58 (78)	41 (79)	
	NYHA IV	18 (14)	13 (18)	5 (10)	
6 months post CRT	NYHA I	13 (10)	13 (18)	0 (0)	fisher's exact testp<0.0001
	NYHA II	50 (40)	47 (64)	3 (6)	
	NYHA III	46 (37)	12 (16)	34 (65)	
	NYHA IV	17 (14)	2 (3)	15 (29)	

There was as a significant shift of patients from NYHA class IV and III to lower classes in patients responding to CRT. Abbreviations: LVEF = left ventricular ejection fraction; CRT = cardiac resynchronization therapy; NYHA class = New York Heart Association functional heart failure classification;

* = p<0.0001 for comparison against baseline.

### 3. Follow-up of responders vs. non-responders

The mean duration of follow-up was 28±14 months (range 9 to 48 month). There were 10 (8%) deaths in our whole collective. Of those 3 occurred in the responder group and were all due to non-cardiac causes, the remaining 7 deaths occurred in the non-responder group and were all attributed to worsening heart failure. There were 8 hospitalizations due to worsening heart failure among 7 Patients in the responder group (11%) and 43 such hospitalizations among 22 patients in the non-responder group (42%). There were no heart transplantations in the responder group whereas there were 2 transplantations among the non-responders.

### 4. Incidence and therapy of ventricular arrhythmias

**Figure 2:**
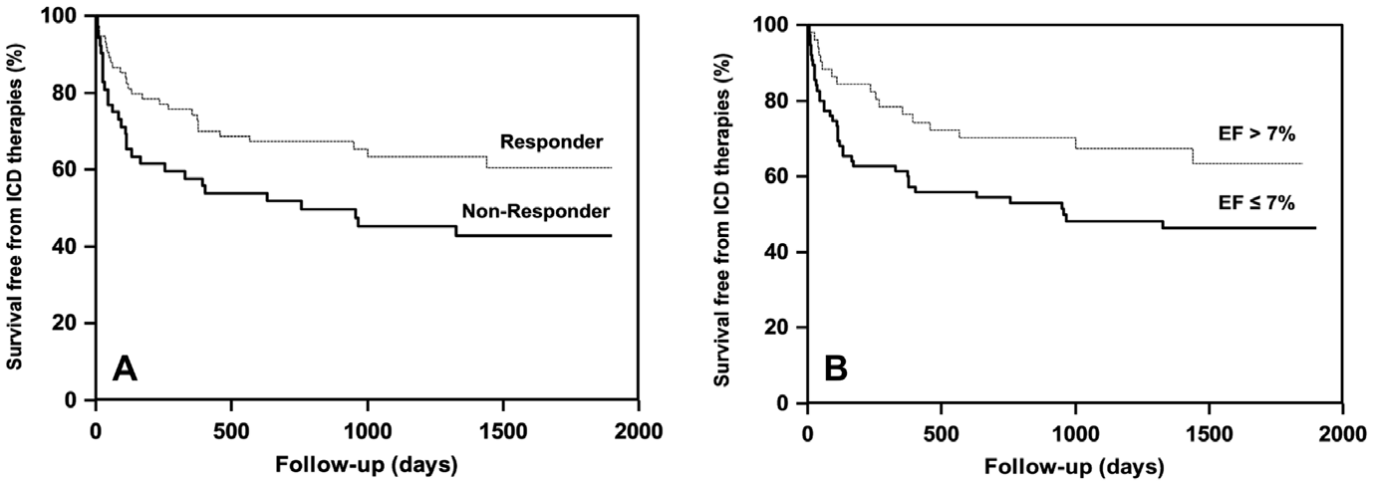
Kaplan-Meier curves of survival free from appropriate implantable cardioverter-defibrillator (ICD) therapy for (A) responders (composite end-point of LVEF increase >10% and/or reduction of 1 NYHA class) and non-responders to resynchronization therapy and (B) in patients with improvement of left ventricular ejection fraction (LVEF) by more respectively 7 or less percent at 6 month after device implantation (as determined by ROC as analysis). In themselves the survival curves illustrate the significant impact of response to CRT (p = 0.0061) as well as the development of LVEF under CRT (p = 0.0168) on the occurrence of ventricular arrhythmia.

**Figure 3:**
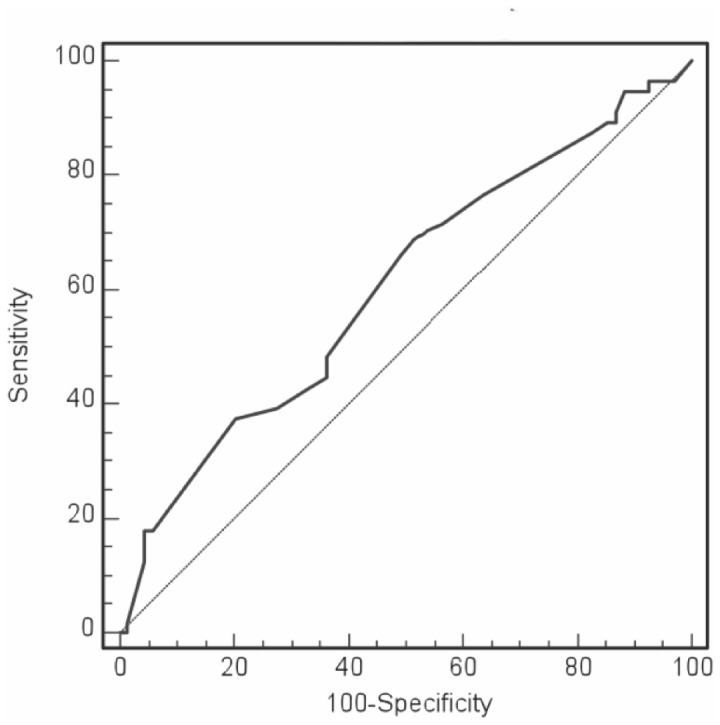
ROC analysis demonstrating the sensitivity and specificity for an increase in EF by >7% as a predictor of ICD therapy (area under the curve = 0.606; standard error = 0.0503; 95% confidence interval = 0.515–0.692; p = 0.0352).

Patients in the responder group received less appropriate ICD therapy than patients in the non-responder group (35% vs. 58%, log-rank X^2^ = 7.5, p<0.0061, Figure 2A). At the end of follow-up, the time between implant and first appropriate ICD therapy was comparable in both groups (19±13 months vs. 15±14 months, p = 0.09). Furthermore, the cycle length of the first ventricular arrhythmia triggering ICD therapy was the same in both groups (responders: 301±48 ms; non-responders: 290±54 ms, p = 0.43). The likelihood of arrhythmia events decreased with increasing LVEF during follow-up (40% vs. 58%, log-rank X^2^ = 5.7, p<0.0168). By employing ROC analysis an EF increase by >7% during CRT was determined as a significant cut-off value (Figure 3) to distinguish individuals with a lower risk of VA occurrence (Figure 2B). The group of patients with secondary preventive ICD-implantation had greater quota of individuals receiving appropriate ICD therapies (n = 20, 71%) than the group of patients with primary preventive indication (n = 36, 37%, p = 0.0002). There was no significant difference in the incidence of appropriate ICD therapies between in patients with ischemic or non-ischemic heart failure.

We also conducted a subgroup analysis comparing non-responders with first VA event in the initial 6 months of CRT (early events) against with first event beyond this period (late events). In this analysis patients with early events had a lesser increase in LVEF during the first 6 months (11.7±27.9% vs. 5.9±24.1%), although the number of patients in those groups was small (n = 28 vs. 24) and the effect was statistically not significant (p = 0.435).

### 5. Predictors of ICD therapy

Cox Regression analysis of clinical baseline parameters indicates that patients on therapy with an ACE inhibitor or an angiotensin receptor antagonist, as well as females a significantly have a reduced risk of VA (HR = 0.24–0.47, p<0.05). Also response to CRT therapy by 6 months after implantation is associated with an reduced risk for VA (HR = 0.53, p<0.05). Secondary preventive indication for device implantation is associated with a significantly higher risk of such events (HR = 3.13, p<0.05) (Table 3).

**Table 3:** Cox Regression analysis of the correlation of selected patient characteristics with the incidence of ventricular arrhythmias (VA) during cardiac resynchronization therapy (CRT).

	b	SE	P	Exp(b)	95% CI of Exp(b)
age >65 years	0.1216	0.2968	0.6819	1.1294	0.6331 to 2.0148
female sex	−0.7467	0.3538	0.03483	0.4739	0.2377 to 0.9448
CAD	−0.04497	0.3125	0.8856	0.9560	0.5198 to 1.7584
secondary preventive	1.1420	0.3203	0.00036	3.1329	1.6777 to 5.8503
ACEI/ATR antagonist	−1.5886	0.6600	0.01609	0.2402	0.05664 to 0.7397
betablocker	−0.4194	0.5329	0.4312	0.6574	0.2326 to 1.8584
digitalis	0.2583	0.3081	0.4019	1.2947	0.7100 to 2.3610
spironolactone	0.16669	0.4093	0.6835	1.1816	0.5319 to 2.6248
responder at 6 months	−0.6370	0.2959	0.03133	0.5289	0.2970 to 0.9417

Patients on therapy with an ACE inhibitor or an angiotensin receptor antagonist, as well as females and responders to CRT show a significantly risk of VA (HR = 0.24–0.53, p<0.05). Secondary preventive indication for device implantation is associated with a significantly higher risk of such events (HR = 3.13, p<0.05). Abbreviations: b = beta, SE = standard error, P = p-value, Exp(b) = hazard ratio (HR), CI = confidence interval.

### 6. Inappropriate ICD therapy

Twelve patients (9.5%) experienced inappropriate ICD shocks. The trigger for inappropriate therapy was atrial arrhythmia in 3 patients, sinus tachycardia in 1 patient, T-wave oversensing in 7 patients, and sensing of diaphragm potentials in 1 patient. Of these 12 patients 7 belonged to the responder group and 5 to the non-responder group, representing 9.5 respectively 9.6% of each group (p = 1.0).

## Discussion

The key findings of this study are: (1.) the incidence of VA is reduced in patients responding to CRT during long-term follow-up. (2.) An increase of LVEF during the first 6 months after commencement of therapy goes along with a decreased incidence VA.

Although there is some evidence favoring antiarrhythmic effects of CRT [4], [31], [32], it's impact on arrhythmia susceptibility is not fully understood with multiple studies providing differing outcomes. Some studies reported a decrease of the number of VA after CRT [33]–[35]; however others reported the opposite [7], [36]. One meta-analysis of large randomized CRT trials found no significant effect of CRT on sustained VA occurrence compared with ICD therapy only [8]. Additionally, a recent meta-analysis including data from the extension phase of Cardiac Resynchronization-Heart Failure (CARE-HF) provided evidence against a benefit of CRT alone on risk of sudden cardiac death (SCD) [37].

In a recent study Lin et al. [38] could not determine any benefit of CRT on the occurrence of VA in a collective of patients undergoing an upgrade from ICD to CRT-D. This might be due to the relatively small sample size and more importantly to the preselection of the patients. All patients included in this study were previously implanted with an ICD, demonstrating a markedly higher risk for VA. Our patient collective in contrast included patients on an “all comers” basis and thus was more heterogenous.

The time course of VA incidence in our study appears to be in line with reports from other investigators, delineating a period from 3 to 12 months after implantation in which electrical reverse remodeling seems to occur [39], [40]. This seems to apply to responders as well as to non-responders, implying that even in non-responders there seems to be some consequence of CRT - just not to the extent as in responders.

We emphasize that the population of our uncontrolled non-randomized observational study is smaller than that included in recent hallmark trials looking for benefit from CRT responding. However, our study design pursued a different aim and was conducted to investigate the incidence of VA in association to improved cardiac function in daily clinical practice.

Contradictory to evidence from previous studies [41], in the presented COX regression analysis of baseline patient characteristics treatment with beta-blockers had no significant effect on VA incidence (HR = 0.657, p = 0.431), This might be in part due to the overpowering impact of other factors in this statistical model. In addition, relatively low beta-blocker dosing, due to adverse effects [31], might be of importance but remains speculative.

Also we chose a definition of reverse remodeling that diverged in some points from the definition used in other current studies. This might impede the comparability of our results.

For the assessment of functional myocardial remodeling under CRT we monitored the development of LVEF during patient follow-up visits. Although LVESV has been shown to be a good indicator of reverse remodeling in previous publications, LVEF was chosen as it is well established and can be measured with comparably low intra- and inter-observer variation during routine echocardiography [42]. Nevertheless this might limit comparability of the presented data.

Defining a positive response to CRT is still crucial. Defining a response as >10% increase of LVEF and/or an improvement of NYHA functional class by ≥1 may limit the number of patients fulfilling these criteria. Also there exist more elaborate classifications of HF, which might aid in the better discrimination of HF stages (e.g. Metra et al. [43]).

However this cut-off is well established in the literature and the observed responder rate of about 70% in our patient population with an LVEF improvement by 7% is in line with various reports [44], [45]. In another recent publication Ypenburg et al. nicely demonstrated that a response to CRT, defined as an improvement in NYHA functional class, was associated with a lower risk of receiving ICD therapy [37], [46].

Herein, we provide evidence during a longer follow-up that improved systolic LV function is associated with a reduced incidence of VA.

Importantly, a possible explanation for the relatively high number of patients with adequate ICD interventions might be early detection and first-line treatment of VA with ATP that may be in fact nsVT. Therefore, ICD therapy would not be a reliable surrogate for SCD, because some VA would likely have terminated spontaneously in the absence of the ICD [7]. However, to determine the risk of sudden cardiac death was beyond the scope of the present study.

### 5. Conclusion

The present findings suggest that improvement of LVEF under CRT in patients with moderate-to-severe heart failure and left bundle branch block, apart from hemodynamic benefits, goes along with a decreased incidence of ventricular arrhythmias.

## Supporting Information

Supplement S1 **Table containing patient characteristics at time of implantation (physiological parameters, prevalence and discrimination of AF types, mitral regurgitation, conduction disorders, indication for ICD therapy and history of ventricular tachycardia or fibrillation before implantation), for responders and non-responders (p-values are given for comparison of both.**
(DOCX)

Supplement S2 **Figure 1** **. Ding cardiovascular risk factors and history of coronary revascularisation for responders and non-responders (p-values are given for comparison of both groups).**
(DOCX)

Supplement S3 **Table containing medication (heart failure therapy, antiarrhythmic agents, anticoagulation) at time of implantation for responders and non-responders (p-values are given for comparison of both groups).**
(DOCX)

## References

[1] Linde C, Ellenbogen K, McAlister FA (2012) Cardiac resynchronization therapy (CRT): Clinical trials, guidelines, and target populations. Heart Rhythm 9: S3–S13. 22521934 10.1016/j.hrthm.2012.04.026

[2] Barsheshet A, Wang PJ, Moss AJ, Solomon SD, Al-Ahmad A, et al (2011) Reverse remodeling and the risk of ventricular tachyarrhythmias in the MADIT-CRT (Multicenter Automatic Defibrillator Implantation Trial-Cardiac Resynchronization Therapy). J Am Coll Cardiol 57: 2416–2423. 21658562 10.1016/j.jacc.2010.12.041

[3] Di Biase L, Gasparini M, Lunati M, Santini M, Landolina M, et al (2008) Antiarrhythmic effect of reverse ventricular remodeling induced by cardiac resynchronization therapy: the InSync ICD (Implantable Cardioverter-Defibrillator) Italian Registry. J Am Coll Cardiol 52: 1442–1449. 19017510 10.1016/j.jacc.2008.07.043

[4] Arya A, Haghjoo M, Dehghani MR, Alasti M, Alizadeh H, et al (2005) Effect of cardiac resynchronization therapy on the incidence of ventricular arrhythmias in patients with an implantable cardioverter-defibrillator. Heart Rhythm 2: 1094–1098. 16188588 10.1016/j.hrthm.2005.07.007

[5] Kurita T, Noda T, Aiba T, Nakajima I, Shimizu W, et al (2011) Cardiac resynchronization therapy to prevent life-threatening arrhythmias in patients with congestive heart failure. J Electrocardiol 44: 736–741. 22018488 10.1016/j.jelectrocard.2011.09.002

[6] Fish JM, Brugada J, Antzelevitch C (2005) Potential proarrhythmic effects of biventricular pacing. J Am Coll Cardiol 46: 2340–2347. 16360069 10.1016/j.jacc.2005.08.035 PMC1474835

[7] Guerra JM, Wu J, Miller JM, Groh WJ (2003) Increase in ventricular tachycardia frequency after biventricular implantable cardioverter defibrillator upgrade. J Cardiovasc Electrophysiol 14: 1245–1247. 14678142 10.1046/j.1540-8167.2003.03303.x

[8] Bradley DJ, Bradley EA, Baughman KL, Berger RD, Calkins H, et al (2003) Cardiac resynchronization and death from progressive heart failure: a meta-analysis of randomized controlled trials. JAMA 289: 730–740. 12585952 10.1001/jama.289.6.730

[9] Singh JP, Gras D (2012) Biventricular pacing: current trends and future strategies. Eur Heart J 33: 305–313. 21951629 10.1093/eurheartj/ehr366

[10] St John Sutton M, Ghio S, Plappert T, Tavazzi L, Scelsi L, et al (2009) Cardiac resynchronization induces major structural and functional reverse remodeling in patients with New York Heart Association class I/II heart failure. Circulation 120: 1858–1865. 19858419 10.1161/CIRCULATIONAHA.108.818724

[11] Ukkonen H, Sundell J, Knuuti J (2008) Effects of CRT on myocardial innervation, perfusion and metabolism. Europace 10 Suppl 3: iii114–iii117. 18955392 10.1093/europace/eun228

[12] Sachse FB, Torres NS, Savio-Galimberti E, Aiba T, Kass DA, et al (2012) Subcellular structures and function of myocytes impaired during heart failure are restored by cardiac resynchronization therapy. Circ Res 110: 588–597. 22253411 10.1161/CIRCRESAHA.111.257428 PMC3299196

[13] Kass DA (2009) Pathobiology of cardiac dyssynchrony and resynchronization. Heart Rhythm 6: 1660–1665. 19879547 10.1016/j.hrthm.2009.08.017 PMC3255324

[14] Aiba T, Tomaselli G (2012) Electrical remodeling in dyssynchrony and resynchronization. J Cardiovasc Transl Res 5: 170–179. 22271011 10.1007/s12265-012-9348-9

[15] Chakir K, Daya SK, Aiba T, Tunin RS, Dimaano VL, et al (2009) Mechanisms of enhanced beta-adrenergic reserve from cardiac resynchronization therapy. Circulation 119: 1231–1240. 19237665 10.1161/CIRCULATIONAHA.108.774752 PMC2850078

[16] Knappe D, Pouleur AC, Shah AM, Cheng S, Uno H, et al (2011) Dyssynchrony, contractile function, and response to cardiac resynchronization therapy. Circ Heart Fail 4: 433–440. 21602574 10.1161/CIRCHEARTFAILURE.111.962902

[17] Chan CP, Zhang Q, Yip GW, Fung JW, Lam YY, et al (2008) Relation of left ventricular systolic dyssynchrony in patients with heart failure to left ventricular ejection fraction and to QRS duration. Am J Cardiol 102: 602–605. 18721520 10.1016/j.amjcard.2008.04.032

[18] Butter C, Wellnhofer E, Seifert M, Schlegl M, Hoersch W, et al (2006) Time course of left ventricular volumes in severe congestive heart failure patients treated by optimized AV sequential left ventricular pacing alone–a 3-dimensional echocardiographic study. Am Heart J 151: 115–123. 16368302 10.1016/j.ahj.2005.02.047

[19] Penicka M, Bartunek J, De Bruyne B, Vanderheyden M, Goethals M, et al (2004) Improvement of left ventricular function after cardiac resynchronization therapy is predicted by tissue Doppler imaging echocardiography. Circulation 109: 978–983. 14769701 10.1161/01.CIR.0000116765.43251.D7

[20] Ypenburg C, van Erven L, Bleeker GB, Bax JJ, Bootsma M, et al (2006) Benefit of combined resynchronization and defibrillator therapy in heart failure patients with and without ventricular arrhythmias. J Am Coll Cardiol 48: 464–470. 16875970 10.1016/j.jacc.2006.04.072

[21] Daubert JC, Saxon L, Adamson PB, Auricchio A, Berger RD, et al (2012) 2012 EHRA/HRS expert consensus statement on cardiac resynchronization therapy in heart failure: implant and follow-up recommendations and management. Heart Rhythm 9: 1524–1576. 22939223 10.1016/j.hrthm.2012.07.025

[22] McMurray JJ, Adamopoulos S, Anker SD, Auricchio A, Böhm M, et al (2012) ESC Guidelines for the diagnosis and treatment of acute and chronic heart failure 2012: The Task Force for the Diagnosis and Treatment of Acute and Chronic Heart Failure 2012 of the European Society of Cardiology. Developed in collaboration with the Heart Failure Association (HFA) of the ESC. Eur Heart J 33: 1787–1847. 22611136 10.1093/eurheartj/ehs104

[23] León AR, Delurgio DB, Mera F (2005) Practical approach to implanting left ventricular pacing leads for cardiac resynchronization. J Cardiovasc Electrophysiol 16: 100–105. 15673400 10.1046/j.1540-8167.2005.04600.x

[24] Bax JJ, Abraham T, Barold SS, Breithardt OA, Fung JW, et al (2005) Cardiac resynchronization therapy: Part 2–issues during and after device implantation and unresolved questions. J Am Coll Cardiol 46: 2168–2182. 16360043 10.1016/j.jacc.2005.09.020

[25] Gehi AK, Mehta D, Gomes JA (2006) Evaluation and management of patients after implantable cardioverter-defibrillator shock. JAMA 296: 2839–2847. 17179461 10.1001/jama.296.23.2839

[26] Koneru JN, Swerdlow CD, Wood MA, Ellenbogen KA (2011) Minimizing inappropriate or “unnecessary” implantable cardioverter-defibrillator shocks: appropriate programming. Circ Arrhythm Electrophysiol 4: 778–790. 22010117 10.1161/CIRCEP.110.961243

[27] Meyer C, Schueller P, Rodenbeck A, Hennersdorf M, Merx M, et al (2009) Primary and secondary prevention of ventricular arrhythmias in dilated cardiomyopathy: nonsustained, sustained, and incessant. Int Heart J 50: 741–751. 19952471 10.1536/ihj.50.741

[28] Lang RM, Bierig M, Devereux RB, Flachskampf FA, Foster E, et al (2005) Recommendations for chamber quantification: a report from the American Society of Echocardiography's Guidelines and Standards Committee and the Chamber Quantification Writing Group, developed in conjunction with the European Association of Echocardiography, a branch of the European Society of Cardiology. J Am Soc Echocardiogr 18: 1440–1463. 16376782 10.1016/j.echo.2005.10.005

[29] Pouleur AC, Knappe D, Shah AM, Uno H, Bourgoun M, et al (2011) Relationship between improvement in left ventricular dyssynchrony and contractile function and clinical outcome with cardiac resynchronization therapy: the MADIT-CRT trial. Eur Heart J 32: 1720–1729. 21609974 10.1093/eurheartj/ehr185

[30] Steffel J, Milosevic G, Hürlimann A, Krasniqi N, Namdar M, et al (2011) Characteristics and long-term outcome of echocardiographic super-responders to cardiac resynchronisation therapy: ‘real world’ experience from a single tertiary care centre. Heart 97: 1668–1674. 21821856 10.1136/heartjnl-2011-300222

[31] Kiès P, Bax JJ, Molhoek SG, Bleeker GB, Zeppenfeld K, et al (2005) Effect of cardiac resynchronization therapy on inducibility of ventricular tachyarrhythmias in cardiac arrest survivors with either ischemic or idiopathic dilated cardiomyopathy. Am J Cardiol 95: 1111–1114. 15842986 10.1016/j.amjcard.2005.01.029

[32] Aiba T, Tomaselli GF (2010) Electrical remodeling in the failing heart. Curr Opin Cardiol 25: 29–36. 19907317 10.1097/HCO.0b013e328333d3d6 PMC2855498

[33] Higgins SL, Yong P, Sheck D, McDaniel M, Bollinger F, et al (2000) Biventricular pacing diminishes the need for implantable cardioverter defibrillator therapy. Ventak CHF Investigators. J Am Coll Cardiol 36: 824–827. 10987605 10.1016/s0735-1097(00)00795-6

[34] Anand IS, Carson P, Galle E, Song R, Boehmer J, et al (2009) Cardiac resynchronization therapy reduces the risk of hospitalizations in patients with advanced heart failure: results from the Comparison of Medical Therapy, Pacing and Defibrillation in Heart Failure (COMPANION) trial. Circulation 119: 969–977. 19204305 10.1161/CIRCULATIONAHA.108.793273

[35] Lellouche N, De Diego C, Boyle NG, Wiener I, Akopyan G, et al (2011) Relationship between mechanical and electrical remodelling in patients with cardiac resynchronization implanted defibrillators. Europace 13: 1180–1187. 21486911 10.1093/europace/eur106 PMC3148818

[36] Di Cori A, Bongiorni MG, Arena G, Soldati E, Giannola G, et al (2005) New-onset ventricular tachycardia after cardiac resynchronization therapy. J Interv Card Electrophysiol 12: 231–235. 15875116 10.1007/s10840-005-0338-6

[37] Cleland JG, Daubert JC, Erdmann E, Freemantle N, Gras D, et al (2006) Longer-term effects of cardiac resynchronization therapy on mortality in heart failure [the CArdiac REsynchronization-Heart Failure (CARE-HF) trial extension phase]. Eur Heart J 27: 1928–1932. 16782715 10.1093/eurheartj/ehl099

[38] Lin G, Rea RF, Hammill SC, Hayes DL, Brady PA (2008) Effect of cardiac resynchronisation therapy on occurrence of ventricular arrhythmia in patients with implantable cardioverter defibrillators undergoing upgrade to cardiac resynchronisation therapy devices. Heart 94: 186–190. 17761506 10.1136/hrt.2007.118372

[39] Kronborg MB, Nielsen JC, Mortensen PT (2010) Electrocardiographic patterns and long-term clinical outcome in cardiac resynchronization therapy. Europace 12: 216–222. 19915182 10.1093/europace/eup364

[40] Boriani G, Biffi M, Martignani C, Ziacchi M, Saporito D, et al (2006) Electrocardiographic remodeling during cardiac resynchronization therapy. Int J Cardiol 108: 165–170. 15923048 10.1016/j.ijcard.2005.04.029

[41] Boriani G, Diemberger I, Valzania C, Biffi M, Martignani C, et al (2010) Role of drugs and devices in patients at risk of sudden cardiac death. Fundam Clin Pharmacol 24: 575–594. 20608989 10.1111/j.1472-8206.2010.00853.x

[42] Jenkins C, Moir S, Chan J, Rakhit D, Haluska B, et al (2009) Left ventricular volume measurement with echocardiography: a comparison of left ventricular opacification, three-dimensional echocardiography, or both with magnetic resonance imaging. Eur Heart J 30: 98–106. 18997179 10.1093/eurheartj/ehn484

[43] Metra M, Ponikowski P, Dickstein K, McMurray JJ, Gavazzi A, et al (2007) Advanced chronic heart failure: A position statement from the Study Group on Advanced Heart Failure of the Heart Failure Association of the European Society of Cardiology. Eur J Heart Fail 9: 684–694. 17481947 10.1016/j.ejheart.2007.04.003

[44] Linde C, Leclercq C, Rex S, Garrigue S, Lavergne T, et al (2002) Long-term benefits of biventricular pacing in congestive heart failure: results from the MUltisite STimulation in cardiomyopathy (MUSTIC) study. J Am Coll Cardiol 40: 111–118. 12103264 10.1016/s0735-1097(02)01932-0

[45] Higgins SL, Hummel JD, Niazi IK, Giudici MC, Worley SJ, et al (2003) Cardiac resynchronization therapy for the treatment of heart failure in patients with intraventricular conduction delay and malignant ventricular tachyarrhythmias. J Am Coll Cardiol 42: 1454–1459. 14563591 10.1016/s0735-1097(03)01042-8

[46] Ypenburg C, van Bommel RJ, Borleffs CJ, Bleeker GB, Boersma E, et al (2009) Long-term prognosis after cardiac resynchronization therapy is related to the extent of left ventricular reverse remodeling at midterm follow-up. J Am Coll Cardiol 53: 483–490. 19195605 10.1016/j.jacc.2008.10.032

