# Supplementary material for: Improvement of Left Ventricular Function under Cardiac Resynchronization Therapy Goes along with a Reduced Incidence of Ventricular Arrhythmia
Source: PLoS One. 2012 Nov 12;7(11):e48926. doi: 10.1371/journal.pone.0048926 (PMC3495960; doi:10.1371/journal.pone.0048926)
Supplement: Supplement S1 — Table containing patient characteristics at time of implantation (physiological parameters, prevalence and discrimination of AF types, mitral regurgitation, conduction disorders, indication for ICD therapy and history of ventricular tachycardia or fibrillation before implantation), for responders and non-responders (p-values are given for comparison of both. (DOCX) [file pone.0048926.s001.docx]

| **Variables** | **All Patients** (n=126) | **Responder** (n=74) (59%) | **Non-Responder** (n=52) (41%) | **p_value** |
| --- | --- | --- | --- | --- |
| Females | 41 (33%) | 25 (34%) | 16 (31%) | 0.847 |
| Age (years) | 64±11 | 64±10 | 64±12 | 0.537 |
| BMI (kg/m^2^) | 27±4 | 28±4 | 26±4 | 0.462 |
| Follow-Up (months) | 28±14 | 26±14 | 28±14 | 0.482 |
| QRS-interval (in ms) | 159±22 | 160±19 | 158±24 | 0.401 |
| Rhythm: |  |  |  |  |
| Sinus rhythmus | 106 (84%) | 63 (85%) | 43 (83%) | 0.806 |
| Paroxysmal AF | 4 (3%) | 2 (3%) | 2 (4%) | 1.0 |
| Persistent AF | 4 (3%) | 2 (3%) | 2 (4%) | 1.0 |
| Permanent AF | 12 (10%) | 7 (9%) | 5 (10%) | 1.0 |
| Newly diagnosed AF during CRT | 24 (19%) | 11 (15%) | 13 (25%) | 0.173 |
| Mitral regurgitation ≥ II° | 43 (34%) | 24 (32%) | 19 (37%) | 0.704 |
| LBBB | 115 (91%) | 69 (93%) | 46 (88%) | 0.359 |
| RBBB | 9 (7%) | 6 (8%) | 3 (6%) | 0.735 |
| AV-block I° | 15 (12%) | 6 (8%) | 9 (17%) | 0.162 |
| AV-block II° | 6 (5%) | 2 (3%) | 4 (8%) | 0.229 |
| AV-block III° | 3 (2%) | 1 (1%) | 2 (4%) | 0.569 |
| CRT-D-indication |  |  |  |  |
| *Primary-prophylactic:* | 98 | 54 (73%) | 44 (85%) | 0.134 |
| nsVT | 17 (13%) | 10 (14%) | 7 (14%) | 1.0 |
| *Secondary-prophylactic:* | 28 | 20 (27%) | 8 (15%) | 0.134 |
| VT | 17 (13%) | 8 (11%) | 9 (17%) | 0.304 |
| VF | 17 (13%) | 9 (12%) | 8 (15%) | 0.608 |
