# Supplementary material for: Improvement of Left Ventricular Function under Cardiac Resynchronization Therapy Goes along with a Reduced Incidence of Ventricular Arrhythmia
Source: PLoS One. 2012 Nov 12;7(11):e48926. doi: 10.1371/journal.pone.0048926 (PMC3495960; doi:10.1371/journal.pone.0048926)
Supplement: Supplement S2 — Figure 1 . Ding cardiovascular risk factors and history of coronary revascularisation for responders and non-responders (p-values are given for comparison of both groups). (DOCX) [file pone.0048926.s002.docx]

| **Variables** | **All patients** (n=126) | **Responder** (n=74) (59%) | **Non-Responder** (n=52) (41%) | **P_value** |
| --- | --- | --- | --- | --- |
| CRF: |  |  |  |  |
| Diabetes | 44 (35%) | 30 (41%) | 14 (27%) | 0.132 |
| Dyslipoproteinemia | 86 (68%) | 51 (69%) | 35 (67%) | 0.849 |
| Family history | 36 (29%) | 21 (28%) | 15 (29%) | 1.0 |
| Hyperuricemia | 63 (50%) | 39 (53%) | 24 (46%) | 0.587 |
| Arterial hypertension | 86 (68%) | 51 (69%) | 35 (67%) | 0.849 |
| Adipositas | 70 (55%) | 46 (62%) | 24 (46%) | 1.0 |
| Nicotin | 78 (62%) | 42 (57%) | 36 (69%) | 0.193 |
| Chronic kidney disease | 77 (61%) | 43 (58%) | 34 (65%) | 0.461 |
| Thyroid dysfunction | 34 (27%) | 20 (27%) | 14 (27%) | 1.0 |
| Coronary  Revascularization |  |  |  |  |
| CABG | 23 (18%) | 10 (14%) | 13 (25%) | 0.108 |
| PTCA | 23 (18%) | 13 (18%) | 10 (19%) | 0.819 |
| Stent | 31 (25%) | 16 (21%) | 15 (29%) | 0.404 |
