# Supplementary material for: Improvement of Left Ventricular Function under Cardiac Resynchronization Therapy Goes along with a Reduced Incidence of Ventricular Arrhythmia
Source: PLoS One. 2012 Nov 12;7(11):e48926. doi: 10.1371/journal.pone.0048926 (PMC3495960; doi:10.1371/journal.pone.0048926)
Supplement: Supplement S3 — Table containing medication (heart failure therapy, antiarrhythmic agents, anticoagulation) at time of implantation for responders and non-responders (p-values are given for comparison of both groups). (DOCX) [file pone.0048926.s003.docx]

| **Variables** | **All patients** (n=126) | **Responder** (n=74) (59%) | **Non-Responder** (n=52) (41%) | **P_vaiue** |
| --- | --- | --- | --- | --- |
| Clopidogrel | 8 (6%) | 4 (5%) | 4 (8%) | 0.717 |
| Phenprocoumon | 74 (59%) | 36 (49%) | 38 (73%) | 0.01 |
| Acetylsalicylic acid | 63 (50%) | 37 (50%) | 26 (50%) | 1.0 |
| ACEI /ATII-antagonists | 122 (97%) | 73 (99%) | 49 (94%) | 0.305 |
| Loop diuretic | 110 (87%) | 62 (84%) | 48 (92%) | 0.184 |
| Thiazide | 48 (38%) | 24 (32%) | 24 (46%) | 0.138 |
| Aldosterone antagonist | 98 (77%) | 59 (80%) | 39 (75%) | 0.664 |
| ß-blocker | 115 (91%) | 67 (91%) | 48 (92) | 1.0 |
| Amiodarone | 34 (27%) | 17 (23%) | 17 (33%) | 0.308 |
| Ca^2+^-antagonist | 7 (5%) | 7 (10%) | 0 (0%) | 0.041 |
| Digitalis | 68 (54%) | 40 (54%) | 28 (54%) | 1.0 |
| Statins | 70 (50%) | 39 (53%) | 31 (60%) | 0.471 |
